# Telehealth Use by Age and Race at a Single Academic Medical Center During the COVID-19 Pandemic: Retrospective Cohort Study

**DOI:** 10.2196/23905

**Published:** 2021-05-20

**Authors:** Jennifer P Stevens, Oren Mechanic, Lawrence Markson, Ashley O'Donoghue, Alexa B Kimball

**Affiliations:** 1 Center for Healthcare Delivery Science Beth Israel Deaconess Medical Center Boston, MA United States; 2 Harvard Medical Faculty Physicians Beth Israel Deaconess Medical Center Boston, MA United States; 3 Information Systems, Beth Israel Deaconess Medical Center Boston, MA United States

**Keywords:** access, barrier, cohort, COVID-19, demographic, equity of care, equity, outpatient, telehealth

## Abstract

**Background:**

During the COVID-19 pandemic, many ambulatory clinics transitioned to telehealth, but it remains unknown how this may have exacerbated inequitable access to care.

**Objective:**

Given the potential barriers faced by different populations, we investigated whether telehealth use is consistent and equitable across age, race, and gender.

**Methods:**

Our retrospective cohort study of outpatient visits was conducted between March 2 and June 10, 2020, compared with the same time period in 2019, at a single academic health center in Boston, Massachusetts. Visits were divided into in-person visits and telehealth visits and then compared by racial designation, gender, and age.

**Results:**

At our academic medical center, using a retrospective cohort analysis of ambulatory care delivered between March 2 and June 10, 2020, we found that over half (57.6%) of all visits were telehealth visits, and both Black and White patients accessed telehealth more than Asian patients.

**Conclusions:**

Our findings indicate that the rapid implementation of telehealth does not follow prior patterns of health care disparities.

## Introduction

During the COVID-19 pandemic, many hospitals reduced in-person ambulatory care. Through payment parity and reductions in administrative barriers, including the Health Insurance Portability and Accountability Act of 1996 waivers by Health and Human Services, physicians rapidly began providing care through telemedicine [1,2].

Our large academic physicians group in Boston, Massachusetts, similarly expanded telehealth in mid-March 2020. This sudden move, however, risked exacerbating care inequity across racial and socioeconomic groups [3,4]. Given potential barriers faced by different populations [5], we investigated whether telehealth use was consistent and equitable across age, race, and gender.

## Methods

### Study Population and Data Source

This study was deemed exempt by the institutional review board at the Beth Israel Deaconess Medical Center. In-person and telehealth visits to outpatient clinics staffed by our practicing faculty, between March 2 and June 10, 2020, for patients aged 18 years were identified and compared to visits to identical clinics during the same period in 2019.

### Study Variables

Patient age (in deciles), self-identified race, and gender were extracted from the electronic health records.

### Statistical Analysis

Statistical tests were performed using Stata SE (version 14.2, StataCorp). In-person and telehealth use were stratified by week and race and compared to the same period in 2019. We conducted similar comparisons by gender and age deciles and explored interaction terms to determine whether different groups of age deciles and race used telehealth more or less than other groups. We further conducted a subgroup analysis of care provided after April 27; after this date, we had more complete documentation of whether the telehealth visit was conducted using video technology or over the telephone. In this subgroup, we explored whether older adults (65 years old) made use of video technology at similar rates as younger patients. We similarly conducted a subgroup analysis during this period to investigate whether non-White patients used video technology at similar rates as White patients.

## Results

Between March 2 and June 10, 2020, a total of 129,844 ambulatory visits were conducted, compared to 180,831 visits during the same period in 2019, which indicates a reduction of 28%. Compared to 2019, visits in 2020 decreased for all racial groups (White patients: 31%, Black patients: 23%, Hispanic patients: 26%, and Asian patients: 39%; *P*<.001).

**Figure 1:**
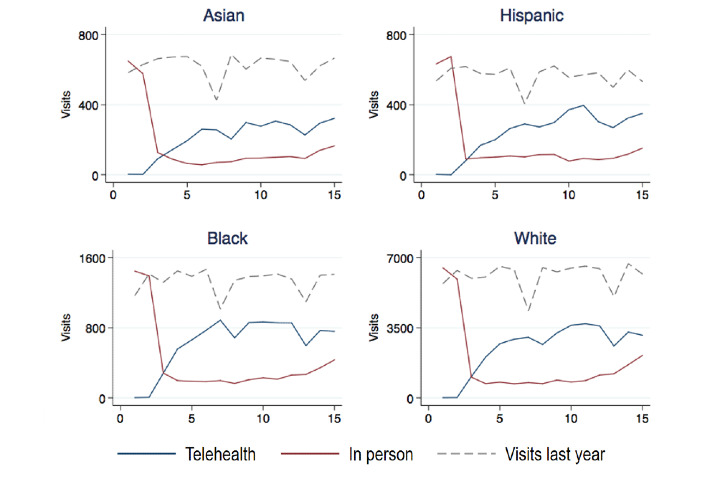
Comparison of outpatient visits in 2020 with those conducted during the same time period in 2019 (dotted line), by race at a single academic health system in Boston, Massachusetts. While all racial groups experienced decreased access to outpatient care owing to the surge in COVID-19 cases from March to June 2020, Asian patients had reduced access to any care (in-person and telehealth) and a decreased rate of access to telehealth services than those of other racial groups. The x-axis represents weeks since March 2020.

Among visits in 2020, a total of 74,846 (57.6%) visits were conducted through telehealth. Overall, Black (n=9414 of 15,423, 61%) and White (n=37,620 of 63,397, 59.3%) patients used telehealth rather than in-person visits at higher rates; Asian patients used telehealth the least (n=3162 of 5661, 55.9%) (Figure 1). Patients with unknown racial designations displayed the lowest rates of telehealth use (*P*<.001).

Older patients accessed more outpatient care than younger patients (*P*<.001) (Table 1). Differences were observed by age and race; differences were most prominent among Asian patients (*P*<.001). Asian and Hispanic patients aged under 65 years used telehealth less than age-matched White patients (*P*<.001), but there was no difference in telehealth usage between Black and White patients under 65 years of age (*P*=.07).

In a subgroup analysis conducted among patients who sought care after April 27, we found that older adults were less likely to use video technology (19% of telehealth visits) rather than the telephone compared to their younger counterparts (26% of telehealth visits; *P*<.001). White patients used video technology significantly more (25% of telehealth visits) than those of all other races (Black patients: 16%, Hispanic patients: 23%, and Asian patients: 17%; *P*<.001).

**Table 1:** Overall usage of in-person and telehealth services from March 2 to June 10, 2020, at a single academic medical center in Boston, Massachusetts, among patients stratified by age and race, recorded at the time of registration (N=129,844).

Parameters				Total		In-person visits		Telehealth visits		*P* value	
**Age (years), n (%)**											<.001
	18-29		11,121 (100)		5179 (46.6)		5942 (53.4)				
	30-39		19,675 (100)		10,234 (52.0)		9441 (48.0)				
	40-49		15,831 (100)		6623 (41.8)		9208 (58.2)				
	50-59		23,238 (100)		9437 (40.6)		13,801 (59.4)				
	60-69		28,120 (100)		11,535 (41.0)		16,585 (59.0)				
	70-79		20,972 (100)		7914 (37.7)		13,058 (62.3)				
	80		10,887 (100)		4076 (37.4)		6811 (62.6)				
**Race, n (%)**											<.001
		Asian	5661 (100)		2499 (44.1)		3162 (55.9)				
		Black	15,423 (100)		6009 (39.0)		9414 (61.0)				
		Hispanic	6243 (100)		2656 (42.5)		3587 (57.5)				
		Other	8499 (100)		3817 (44.9)		4692 (55.1)				
		Unknown	30,621 (100)		14,240 (46.5)		16,381 (53.5)				
		White	63,397 (100)		25,777 (40.7)		37,620 (59.3)				
**Total, n (%)**				129, 844 (100)		54,998 (42.4)		74,846 (57.6)			

^a^N/A: not applicable.

## Discussion

### Principal Findings

Our results indicate that implementation of telehealth does not necessarily exacerbate the inequity in health care access, but it should be monitored carefully. Patients had fewer ambulatory visits of any kind during the COVID-19 pandemic; however, Asian and White patients accessed care less than Black and Hispanic patients compared to baseline. Black and White patients accessed telehealth care more than Hispanic and Asian patients. Patients with an unknown racial or ethnic designation upon registration had the least access to care.

Contrary to concerns that older patients might have difficulty navigating technology [6], our older patients used telehealth more often than younger patients. The potential reasons for this could be that younger patients perceived their clinical needs to be less urgent, had variable awareness of telehealth services, or experienced additional barriers [7]. However, we found that among telehealth users, patients over 65 years of age were less likely to use video technology, which may reflect concerns with technology; these concerns have been explored by other investigators [6,8]. Similarly, Black, Asian, and Hispanic patients were less likely to use video technology than White patients.

### Limitations

The limitations of this study include the use of administrative data and self-reported racial designations; approximately 22% of patients in our study belonged to an unknown racial category, which may limit our ability to draw inferences [9,10]. Further, our documentation of telehealth early in the pandemic was unable to distinguish telephonic and video visits, which may vary across racial and age groups [6].

### Conclusions

In conclusion, after a rapid increase in telehealth use at a single academic medical center in Boston, Massachusetts, we observed variable engagement of our patient population by both race and age in telehealth, but the trends did not mirror previously described patterns of health access disparity.
